# Changing Therapy for Gastrinoma

**DOI:** 10.1155/1998/62935

**Published:** 1998

**Authors:** P. C. Bornman, K. Radebold

**Affiliations:** Department of Surgery University of Cape Town Medical School Observatory 7925 Cape Town South Africa

## Abstract

*Objective*: The author analyzed potential survival determinants in gastrinoma to characterize a possible uniform staging system and to determine whether complete surgical resection improves expected survival.

*Summary and Background Data*: Gastrinoma is an indolent yet malignant neuroendocrine tumor. The associated gastric acid hypersecretion can be controlled medically. Staging of gastrinoma is inconsistent and the role Of surgical resection controversial.

*Methods*: Seventy-four patients with gastrinoma with a minimum 5-year follow-up were assessed. Cox's proportional hazards regression model was used to examine the association of risk factors with survival.

*Results*: The following factors had no effect on survival: age at diagnosis, sex, presence of lymph node metastases, associated multiple endocrine neoplasia, and method of ulcer treatment. The three unique determinants of survival were primary tumor size (relative risk 1.534; *p*<0.0005), liver metastases (relative risk, 2.947; *p*<0.0209), and complete surgical resection (relative risk 0.163; *p*<0.0076). On the basis of these risk factors, a uniform staging system is proposed and predictive survival curves developed.

*Conclusions*: The primary determinants of survival in gastrinoma are the size of the primary tumor and liver metastases. Complete surgical resection reduces mortality, regardless of other factors.


### Changing Therapy for Gastrinoma

### ABSTRACT

*Ellison, E. C., (1995) Forty-year appraisal of gastrinoma: Back to the future. Annals of Surgery; 222, 511–524.*

*Objective:* **The author analyzed potential survival determinants in gastrinoma to characterize a possible uniform staging system and to determine whether complete surgical resection improves expected survival.**
*Summary and Background Data:* **Gastrinoma is an indolent yet malignant neuroendocrine tumor. The associated gastric acid hypersecretion can be controlled medically. Staging of gastrinoma is inconsistent and the role of surgical resection controversial.**
*Methods:* **Seventy-four patients with gastrinoma with a minimum 5-year follow-up were assessed. Cox's proportional hazards regression model was used to examine the association of risk factors with survival.**
*Results:* **The following factors had no effect on survival: age at diagnosis, sex, presence of lymph node metastases, associated multiple endocrine neoplasia, and method of ulcer treatment. The three unique determinants of survival were primary tumor size (relative risk, 1.534; $p = 0.0005$), liver metastases (relative risk, 2.947; $p = 0.0209$), and complete surgical resection (relative risk, 0.163; $p = 0.0076$). On the basis of these risk factors, a uniform staging system is proposed and predictive survival curves developed.**
*Conclusions:* **The primary determinants of survival in gastrinoma are the size of the primary tumor and liver metastases. Complete surgical resection reduces mortality, regardless of other factors.**

*Keywords:* Gastrinoma

### PAPER DISCUSSION

The study of risk factors and their implication on survival in Zollinger-Ellison syndrome (ZES) became possible with the introduction of potent acid suppression medication with few patients now dying from the consequences of high acid secretion. Within a period of months two landmark articles have appeared on the natural history and prognosis of the ZES by Ellison and by Weber *et al.* [1]. The large numbers in both series and the detailed analysis of their data have for the first time allowed a more accurate assessment of the factors determining the prognosis. The analysis of the data in both series were similar with the emphasis on various clinicopathological tumor characteristics such as gastrinoma size, location, metastases to liver or lymph nodes and association to MEN-I.

In his study Ellison divided his patients into two groups consisting of patients treated between 1947–1979 (pre-$H_2$ receptor antagonist era, 40 patients) and 1980 –1994 ($H_2$ receptor antagonist era, 34 patients). He categorized both groups with regard to tumor characteristics, gastric operations, tumor resection and cause of death. The analysis resulted in identifying three risk factors which were associated with survival: liver metastases, tumor size, and resection. On the basis of these risk factors he proposed a staging system for gastrinoma based on primary tumor size and metastases. Tumor location, multicentricity and lymph node metastases were not considered to be independent prognostic factors. He concluded that regardless of the site of the primary tumor the small tumor size and complete surgical resection improved the survival.

Weber *et al.* based their findings on data selected from 185 consecutive patients from the National Institute of Health over a 15 year period. Both series showed remarkable similar figures for patients with MEN-I (Ellison 12% in the $H_2$ receptor antagonist era, Weber 18%), histological confirmation of ZES (82% *vs.* 89%), occurrence of liver metastases (21% *vs.* 24%) and the location of pancreatic (35% in the $H_2$ era *vs.* 30%) and duodenal primaries (26% in the $H_2$ era *vs.* 30%). However, Ellison reported on only 4% (3/74) "lymph node primaries" while Weber found gastrinoma tissue confined only to lymph nodes in 24 (13%) of his 185 patients.

Unlike in the previous publication from the same center Ellison did not analyse the prognostic value of the MEN-I syndrome [2]. In this 1993 publication 20-year survival was appreciably better in patients with the MEN-I syndrome (58% *vs.* 31% without MEN-I syndrome), an observation which is shared by Weber (93% *vs.* 68% 15-year survival, $p = 0.06$). The most logical explanation for this difference is the higher incidence of associated liver metastases in the sporadic group which suggests that the ZE patients harbored more aggressive tumors.

Both authors used various statistical models to determine the significance of tumor location and size of tumor on prognosis. Their results indicate that the anatomical site is not a determining factor. Ellison suggests that tumor location is a confounding co-variable of size and not an independent factor for survival. But with pancreatic tumors being bigger in size when detected and therefore more prone to metastasize to the liver, location still may be an important factor influencing survival.

The two studies agreed that there is a highly significant correlation of primary tumor size, the development of liver metastases and survival. The ultimate prognosis is determined by the presence of liver metastases; when liver metastases were present the 10-year survival was reduced from 90% to about 30%.

Our experience of 7 patients with liver metastases (of a total of 34 patients with gastrinomas) showed that survival can vary within a wide range depending on the biological behaviour of the tumor [3]. It is our impression that the overall prognosis of metastatic gastrinoma to the liver is good, even if multiple metastases are present. Minimal extrahepatic disease seems to be a favourable prognostic factor in these patients [3].

Both authors finally concluded that with size being one of the important factors for survival, surgery has to focus on complete tumor resection. In Ellison's series as well as in others, more than two thirds of all deaths in ZE patients were caused by progression of the disease with the development of metastases [4]. In another study Fraker also emphasizes the importance of tumor resection on the basis of his experience [5]. In his series medically treated patients had a seven fold increased risk of developing liver metastases when compared to patients who had excision of the primary tumor. These observations indicate routine exploratory laparotomy in surgically fit patients with excision of all macroscopic tumor and, when feasible, of liver metastases.

While Ellison's attempt at a new staging system for gastrinomas is plausible, it is doubtful whether the TNM classification is appropriate for endocrine tumors which have a different biological behaviour, particularly with regard to metastases [6]. Since survival is not influenced by lymph node metastases the listed lymph node status in the classification would seem inappropriate. While tumor size is of prognostic importance it is not clear how the author derived the various sizes for primary tumor staging (T1-4). Indeed 10-year survival was similar in patients without liver metastases regardless of tumor size when the primary tumor was resected. Therefore, other than for the presence of liver metastases, this classification is of limited value for surgical decision making or for predicting outcome. Nevertheless, it is to the author's credit that he initiated a new look at a staging system for gastrinoma and other endocrine tumors. These data should stimulate other centers to review their own experience to refine an appropriate and practical classification.

### References

[1] Weber, H. C., Venzon, D. J., Lin, J. T., Fishbein, V. A., Obruch, M., Strader, D. B., Gibril, F., Metz, D. C., Fraker, D. L., Norton, J. A. and Jensen, R. T. (1995). Determinants of metastatic rate and survival in patients with Zollinger–Ellison Syndrome: a prospective long-term study. *Gastroenterology*, **108**, 1637–1649.

[2] Melvin, W. S., Johnson, J. A., Sparks, J., Innes, J. T. and Ellison, E. C. (1993). Long-term prognosis of Zollinger-

Ellison syndrome in multiple endocrine neoplasia. *Surgery*, **114**, 1183 – 1188.
[3] Radebold, K., Borman, P. C., van Wyk, M. E. C., Krige, J. E. J., Mostert, C. and Terblanche, J. (1996). Clinicopathological characteristics and prognosis of liver metastases in patients with Zollinger – Ellison syndrome. *Gastroenterology*, **110**, A1100.
[4] Zollinger, R. M. (1985). Gastrinoma: factors influencing prognosis. *Surgery*, **97**, 49 – 54.
[5] Fraker, D. L., Norton, J. A., Alexander, H. R., Venzon, D. J. and Jensen, R. T. (1994). Surgery in Zollinger – Ellison syndrome alters the natural history of gastrinoma. *Ann Surg*, **220**, 320 – 330.
[6] UICC International Union Against Cancer. TNM classification of malignant tumours (1987). Springer Verlag Berlin Heidelberg New York.

Professor P. C. Bornman
Head: Surgical Gastroenterology and
Gastrointestinal Clinic E23

K. Radebold, M.D.
Consultant Surgeon
Department of Surgery
University of Cape Town Medical School
Observatory 7925
Cape Town
South Africa

### Small-Diameter PTFE Portosystemic Shunts: Portocaval vs Mesocaval

### ABSTRACT

*Paquet, K.-J., Lazar, A., Koussouris, P., Hotzel, B., Gad, H. A., Kuhn, R. and Kalk, J.-F. (1997) Mesocaval interposition shunt with small-diameter polytetrafluoroethylene grafts in sclerotherapy failure, British Journal of Surgery; 82, 199–203.*

**Fifty-seven patients with failed sclerotherapy received a mesocaval interposition shunt with an externally supported, ringed polytetrafluoroethylene prosthesis of either 10 or 12 mm diameter. Thirty-one patients had Child-Pugh grade A disease and 26 grade B; all had a liver volume of 1000 – 2500 ml. Follow-up ranged from 16 months to 6 years 3 months. Three patients (5 per cent) died in the postoperative period. There were two postoperative recurrences of variceal haemorrhage and one recurrent bleed in the second year after surgery. The cumulative shunt patency rate was 95 per cent and the incidence of encephalopathy 9 per cent; the latter was successfully managed by protein restriction and/or lactulose therapy. The actuarial survival rate for the whole group at 6 years was 78 per cent, for those with Child-Pugh grade A 88 per cent and for grade B 67 per cent. Small-lumen mesocaval interposition shunting achieves portal decompression, preserves hepatopetal flow, has a low incidence of shunt thrombosis, prevents recurrent variceal bleeding and is not associated with significant postoperative encephalopathy.**

*Keywords:* Portocaval shunt, mesocaval shunt, Sarfeh shunt, narrow-diameter shunt

### PAPER DISCUSSION

The place of surgical operations in the treatment of portal hypertension has become much more circumscribed. For the most part, initial treatment, both emergency and elective, is non-surgical and involves a direct attack on the varices, either sclerotherapy or banding. Balloon tamponade and vasoactive drugs represent adjuvant treatment only. Surgery is used when sclerotherapy or banding fails. Because of the post-operative incidence of hepatic failure and encephalopathy, end-to-side portacaval shunt was abandoned in favour of operations aimed to maintain prograde portal flow and hepatic perfusion, e.g. distal splenorenal shunt, small-diameter side-to-side portacaval shunts, etc. The value of maintaining hepatic perfusion remains largely theoretical. The operation of mesocaval interposition shunt had the advantage of technical simplicity. However, although the initial results seemed good, long-term outcome was not so optimistic in terms of rebleeding and encephalopathy. Graft thrombosis was common.

